# Advances in Quantitative Trait Analysis in Yeast

**DOI:** 10.1371/journal.pgen.1002912

**Published:** 2012-08-16

**Authors:** Gianni Liti, Edward J. Louis

**Affiliations:** 1 Institute of Research on Cancer and Ageing of Nice (IRCAN), CNRS UMR 7284–INSERM U1081, Faculté de Médecine, Université de Nice Sophia Antipolis, Nice, France; 2 Centre for Genetics and Genomics, Queens Medical Centre, University of Nottingham, Nottingham, United Kingdom; Washington University School of Medicine, United States of America

## Abstract

Understanding the genetic mechanisms underlying complex traits is one of the next frontiers in biology. The budding yeast *Saccharomyces cerevisiae* has become an important model for elucidating the mechanisms that govern natural genetic and phenotypic variation. This success is partially due to its intrinsic biological features, such as the short sexual generation time, high meiotic recombination rate, and small genome size. Precise reverse genetics technologies allow the high throughput manipulation of genetic information with exquisite precision, offering the unique opportunity to experimentally measure the phenotypic effect of genetic variants. Population genomic and phenomic studies have revealed widespread variation between diverged populations, characteristic of man-made environments, as well as geographic clusters of wild strains along with naturally occurring recombinant strains (mosaics). Here, we review these recent studies and provide a perspective on how these previously unappreciated levels of variation can help to bridge our understanding of the genotype-phenotype gap, keeping budding yeast at the forefront of genetic studies. Not only are quantitative trait loci (QTL) being mapped with high resolution down to the nucleotide, for the first time QTLs of modest effect and complex interactions between these QTLs and between QTLs and the environment are being determined experimentally at unprecedented levels using next generation techniques of deep sequencing selected pools of individuals as well as multi-generational crosses.

## Yeast Genetics: Moving Forward

Yeast genetics started over 70 years ago when Ojvind Winge successfully crossed two different strains to create a hybrid that combined desirable traits from both parents [1]. The ability to make crosses and generate segregants led to budding yeast developing into one of the premier genetic models. Winge's basic experiment is also the initial step of any forward genetics approach requiring the generation of a recombinant offspring from genetically diverged strains [2]. However, this route was rarely taken. Instead, *S. cerevisiae* has been used as a workhorse to dissect phenotypes, function, and mechanism, where the effect of mutations, initially generated randomly and then systematically, were screened for specific phenotypes [3]. *S. cerevisiae* became the first eukaryote sequenced and a handful of laboratory strains, mostly related to the sequenced S288c background, have been used universally.

**Figure 1:**
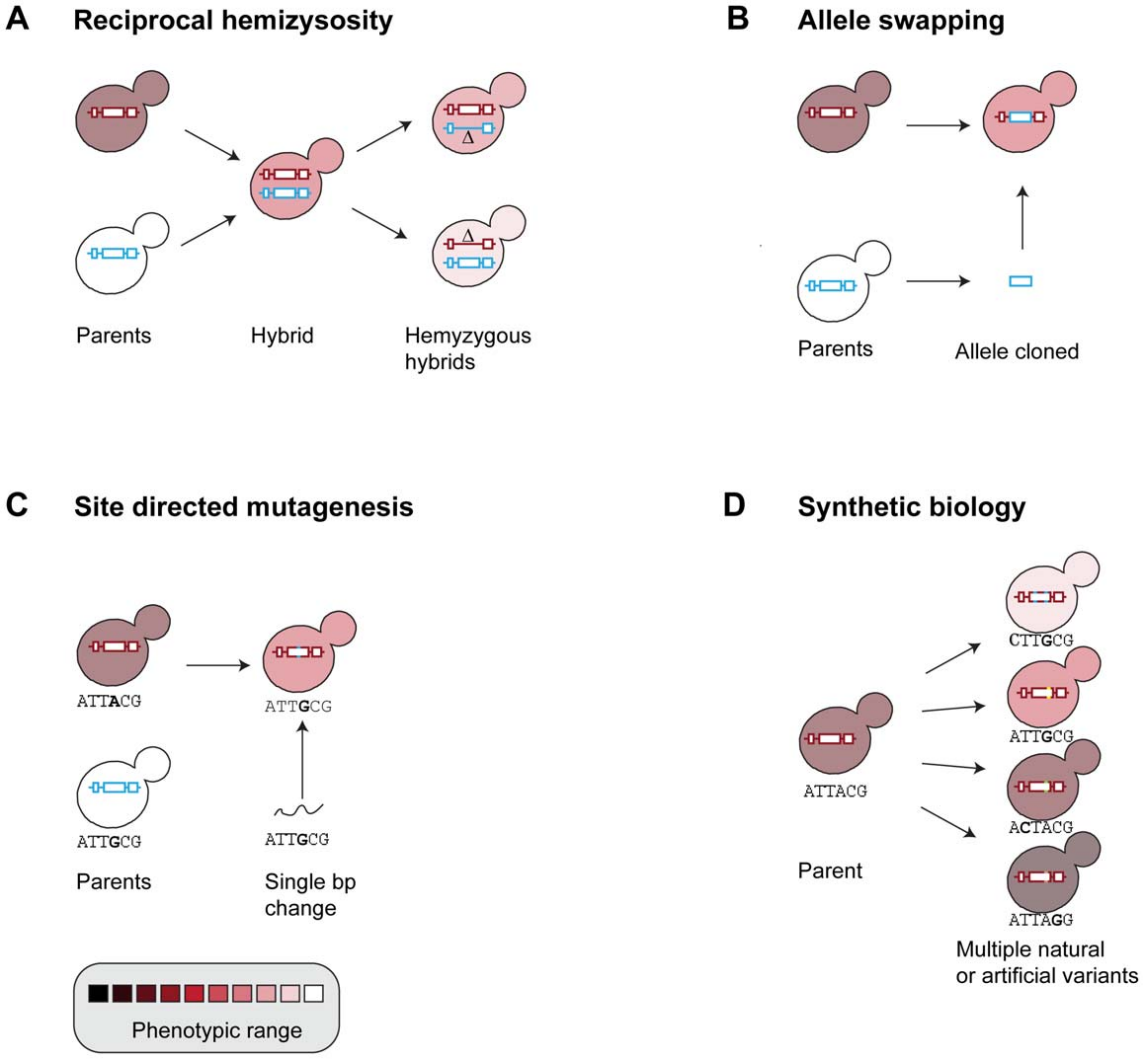
Experimental measures of natural variation. Yeasts offer a unique opportunity to engineer changes to measure the impact of phenotypic variants on traits. (A) Reciprocal hemizygosity has high throughput and can be used to test a large number of candidates. Hybrids that differ only in which of two alleles is present/deleted are compared. Deletion collections of multiple strains will soon be available allowing genome-wide systematic studies using hybrids to test all candidates easily or even for discovery of phenotypic effects directly. (B) Allele swapping is less high throughput but allows testing phenotypic effects of specific alleles in different genetic backgrounds. This is more precise than reciprocal hemizygosity. (C) Site-directed mutagenesis is a rapid and precise way of testing known and novel base changes for phenotypic effects. (D) Synthetic biology has the potential of simultaneously testing multiple variants, both natural or artificial, in a single gene [55] or scattered through the genome [47].

In the past decade this trend has changed and *S. cerevisiae* has emerged as a powerful model for quantitative forward genetics. A series of studies have mostly used two hybrids to generate sets of segregants: the BY/RM hybrid, essentially used to find expression QTLs (eQTL) [4], and the YJM/S288c hybrid, to accurately dissect the architecture of a single trait [5]. The study of eQTLs [4], [6] is conceptually different, generally Mendelian variants segregating that affect expression of a gene in cis or trans depending on whether they are in the regulatory sequence or in a transcription factor, and requires a separate discussion. These studies were instrumental in showing how the biological and genetic features of budding yeast make this organism an ideal model for developing a deeper understanding of quantitative genetics. Some of the principles that emerged from these studies on the complex architecture of traits broadly apply to other organisms. In addition, a simple and elegant approach named reciprocal hemizygosity was devised to validate the effect of one allele over the other in an F1 hybrid [5], and the same principle was successfully exploited in other organisms (Figure 1A) [7]. We now have the exquisite precision in mapping and validating functional variants down to single nucleotide resolution (QTN) (Figure 1B–D). One of the most exhaustive studies identified four QTNs and their interactions that control sporulation (meiosis) efficiency with three of the QTNs being transcription factors [8].

These studies revealed principles regarding the genetic architecture of traits that have since been found to be broadly applicable by other studies. A recurring theme is that many apparent QTLs are actually composed of several linked QTNs. Counterintuitively, some of the QTLs in a linkage group have effects opposite to expectations based on the parental phenotype (transgressive QTLs). Finally, in virtually every cross the phenotypic variation seen in the progeny is greater than that seen between the parents. Together these properties make complex trait analysis in yeast at the same time both more difficult than might be expected and potentially very fruitful with respect to building a general understanding of the genetic architecture of traits in all organisms.

## Population Genomics and Standing Genetic Variation

Early QTL mapping experiments clearly showed the potential of budding yeast as a model in quantitative genetics. However, very little was known about the origin of the strains selected for breeding. Large collections of strains have been isolated from substrates that originate from human activity [9], [10]. In addition to these, other studies reported the isolation of *S. cerevisiae* from multiple niches in wild environments [11], [12]. The genome sequence of several diverse isolates revealed the presence of five genetically diverged clean lineages (populations that are not interbreeding) [13]. The majority of segregating sites in each clean lineage are private and monomorphic within the lineage. Some of these phylogenetic clusters grouped strains isolated from specific human activity (e.g., cultivation from wine and sake production), indicating a potential role of human activity in their evolution and perhaps partial domestication [9]. Half of the sequenced strains have mosaic recombinant genomes (mixed ancestry) originating from outcrosses between the clean lineages and are polymorphic for the majority of segregating sites. Importantly, genome analysis provides convincing evidence that human activity has not resulted in reduced standing variation and access to this reservoir of variability is a great resource for investigating the genetic structure of complex traits [3]. The population structure of *S. cerevisiae*, with several clean lineages and several outbred mosaics, may not be ideal for genome-wide association studies (GWAS), as there is a strong linkage of SNPs on a population level and there may not be enough independently derived mosaic strains to overcome the linkage disequilibrium. However, strains belonging to the clean lineages are ideal for linkage analysis as they have an even distribution of segregating sites across the genome and these polymorphisms have coevolved within a specific genomic context. Resources of genotyped segregants from a grid of crosses are now available [14], [15].

A recurrent problem is that parts of the variation are inaccessible to linkage analysis due to reproductive isolation. The low gamete viability can be attributed to chromosomal rearrangements, sequence divergence, and genetic incompatibilities. For example, one of the *S. cerevisiae* clean lineages, isolated from the Malaysian rain forest, is reproductively isolated from all the other lineages [14]. Initial attempts at using even more genetically distant strains, like populations of *S. paradoxus* from different continents, gave evidence of deleterious interactions perhaps due to partial incompatibility of the highly diverged alleles [16]. This high sequence variation resulted in reduced recombination, making QTL mapping particularly difficult. This reduced recombination also leads to partial reproductive isolation, adding to the difficulty. The problem of reproductive barriers also applies to other model systems. Crosses between *Schiz. pombe* isolates often result in very poor gamete viability (Jurg Baheler, personal communication), and genetic incompatibilities have been observed in *C. elegans* crosses [17]. This problem can be overcome with appropriate nested backcross approaches.

## From Variant to Variation

**Figure 2:**
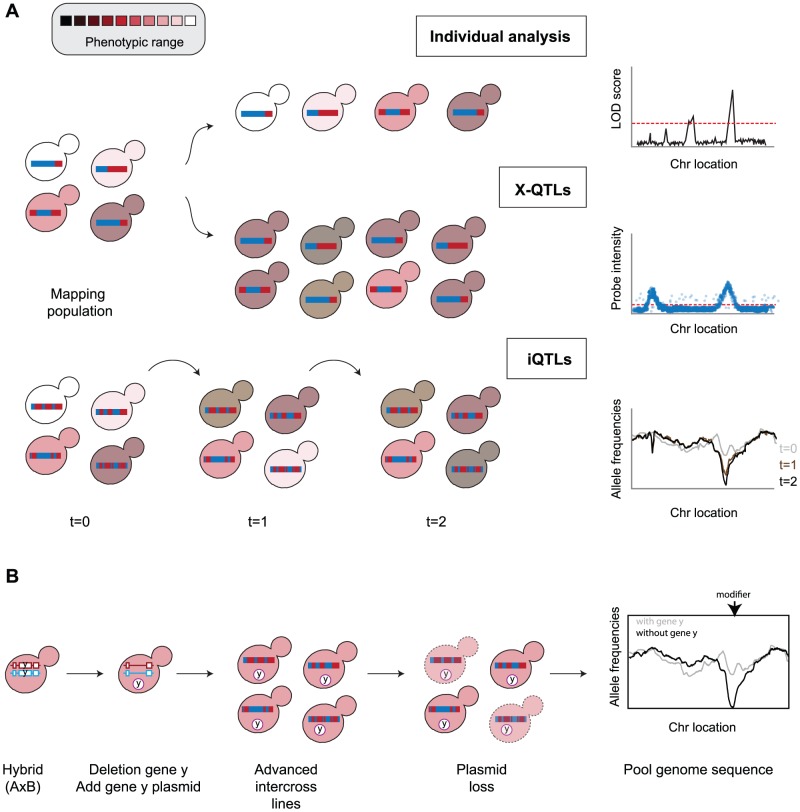
Mapping QTLs and modifiers. (A) QTL mapping has evolved from the classical approach of individual segregant analysis to the X-QTLs and iQTLs approaches with higher mapping sensitivity and resolution. Analysis of time series data in iQTLs allows dynamic monitoring of allele frequency values [56]. (B) A possible approach to map genetic modifiers using iQTLs. A conditional essential gene, *y*, is deleted from its original chromosomal location and maintained on a plasmid. This hybrid is intercrossed multiple times to allow reshuffling of parental genomes. Upon loss of gene *y*, viability relies on the presence of genetic modifier/s, and allelic combinations that result in lethality (dashed cells) will decrease in allele frequency. These modifiers can be detected by comparing allele frequencies of the pool before and after the plasmid loss. When many modifiers are involved, the lethal combinations will be present in low frequency, making them difficult to detect. Further rounds of intercrosses, after loss of gene *y*, will allow reshuffling of alleles and the generation of more cells with unviable combinations.

The effect of causative genetic variants is not only determined by the polymorphisms themselves but is also shaped by genetic and environmental interactions. Many polymorphisms associated with disease risk only cause disease in some individuals [18]. Similarly, the majority of yeast QTLs appear to be context dependent, thus acting only in specific cross combinations or environments [14], [19]. One of several heat tolerant QTLs, for example, is found only in a specific cross out of six pairwise crosses between four parents [13]. This phenomenon is also observed in reverse genetic studies where inactivating the same gene in multiple strains can have a different phenotypic outcome. An interesting example of conditional viability was previously reported for a duplicated pair of histone coding genes, H2A and H2B [20]. Strains can survive deletion of one of the pairs by upregulating the expression of the other, however deletion of the other pair cannot be compensated in this way. Some strains were able to survive this deletion because of a Ty-mediated gene amplification mechanism of the first pair, which allowed the necessary dosage compensation. Yeast again provided a systematic approach, and genome-wide deletion collections in two different strain backgrounds were analyzed for viability [21]. Despite the two strains sharing ancestry (half of their coding genome is identical), 10% of the genes were conditionally essential: lethal in one strain and with no obvious phenotype in the other. Interestingly, the dispensability of these genes depends on several other modifiers, but their mechanisms of interaction are still unknown. These genes can be identified using conventional QTL mapping, and a recent bulk segregant approach appears particularly promising in mapping these (Figure 2).

The surrounding environment provides a second level of QTL regulation. Similar to the genetic background effects, many QTLs are only effective in specific environmental conditions. For example, genes affecting colony morphology are strongly regulated by the nutrients present in the media [22]. Here two gene regulatory networks interact with each other and environmental signals to produce a switch to filamentous growth. Genes residing in the subtelomeric regions are also subject to environmental regulation [23] through environment-responsive transcription factors. Furthermore, the environmental conditions also shape the gene-gene interaction network generating an even more complex interaction (gene×gene×environment). In the example of the four QTNs affecting sporulation [8], the 32 possible combinations of genotypes in both genetic backgrounds were exposed to eight environmental conditions [23]. Clear examples of gene×gene interactions dependent on environment were found. The environmental effect can be direct but also mediated by master regulators such as the chaperone HSP90. Modulation of *HSP90* by temperature and drugs reveals the presence of *HSP90* contingent QTLs [24]. In this case, environment-dependent alleles are indirectly modulated via *HSP90* through protein folding. An intriguing intermediate role for epigenetic polymorphisms has also been proposed [25] where histone mark differences appear enriched in genes with high transcriptional variability.

## The Genomic Landscape of QTLs

One issue with mapping QTLs is complete knowledge of the genomes. In general, subtelomeric regions are incomplete in most genome projects and if complete only so in a high-quality reference genome such as the case of *S. cerevisiae*. As subtelomeres are highly polymorphic with respect to presence/absence and location of genes and sequence, as well as in sequence variation, there is the potential for a great deal of unmappable variation. In budding yeast studies upwards of 25% of QTLs for overall traits have been mapped distal to the last segregating marker [14]. The missing genetic information is, however, only about 8% of the genome. Some of this variation may be due to copy number of genes present in different subtelomeres as has been seen for arsenate resistance [13] but not all will be due to CNV. Not only are QTLs missed by not having complete genomes, they appear to be enriched in the missing regions. Getting a handle on this poorly described part of the genome at the level of variation in a population will be one of the major goals of the next decade.

**Figure 3:**
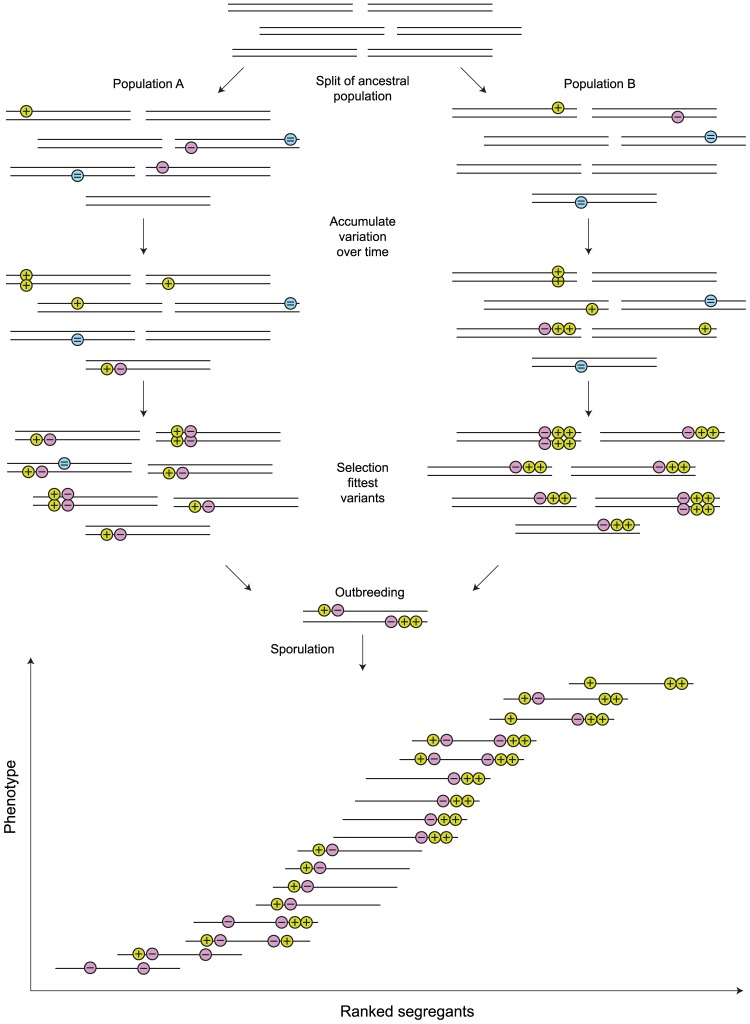
Linked quantitative trait loci (QTLs) can arise through normal population genetic processes. For any given phenotype there are many loci where mutations can have an effect. Different populations will experience mutations in different loci affecting the same phenotype. These mutations can affect a phenotype in a positive (+) or negative (−) way and if nearly neutral ( = ) will remain segregating within a population for awhile. As other mutations occur, advantageous combinations can result with better fitness than either mutation alone or the original parental alleles. Multiple mutations with effects upon a trait will be broken up by recombination if not linked and one or more can therefore be lost. Linked mutations can become fixed as blocks of larger collections of QTLs if the combination of alleles is beneficial. Different populations may evolve different “super”-QTLs, which are revealed when the populations interbreed. Offspring will express a range of phenotypes depending on which QTLs are inherited and how much recombination breaks up the linked groups. Multiple rounds of interbreeding can further break up the linked QTLs revealing individual loci, as illustrated in Figure 2.

One interesting observation that has arisen from the dissection of QTLs responsible for specific traits, in the F1 progeny of a given cross, is that an initial large QTL region turns out to be composed of several linked QTLs. Furthermore these are a mixture of antagonistic QTLs (those alleles with a different effect from their parental origin) and QTLs with the expected phenotypic effect (coinciding with parental phenotype). Two specific studies using the lab reference strain and one other to create F1 progeny mapped a QTL with large effect for heat resistance and sporulation [5], [26]. Upon further dissection of these regions, three and four linked QTLs were determined. In the case of heat resistance, one of three QTLs was antagonistic, and in the case of sporulation, three out of four were antagonistic. Interestingly, a separate study of sporulation QTLs using the same cross [27] did not find the same QTLs, and those found were unlinked. The differences between the studies include mapping approaches but also the phenotype measured. In one case it was sporulation at 24 h and the other at 7 d, showing another level of complexity in the analysis of quantitative traits. A third case has recently been found in ethanol tolerance in an industrial strain with four linked QTLs, one being antagonistic [28]. Linked QTLs have also been observed in Arabidopsis [29] with QTLs affecting growth rate. At first glance this seems unexpected, as one would expect QTLs to have effects in the same direction as exhibited by the parent (in the case of yeast, the parental population is fixed for these variants), and linkage of these QTLs would be improbable due to chance alone. One possibility for linkage is the selective sweep of a beneficial allele carrying less adapted linked alleles by hitchhiking. There is no reason, however, that other loci involved in the trait should be linked, certainly not as often as is observed. An alternative hypothesis is through adaptation. Isolated populations of yeast may be expected to result in linked QTLs of mixed effect in the following manner. It is known from genome-wide forward genetic studies that large numbers of genes can affect a given phenotype. A new mutation, affecting the trait which may be advantageous or not disadvantageous enough to be removed immediately from the population, will be a QTL for a particular phenotype. Additional mutations in that population that affect that phenotype and the fitness of the individuals carrying both, either by compensating a slightly deleterious initial mutation or furthering the advantage of the individual carrying both, will increase in the population if it is fitter than either mutation alone or the original genotype. For *Saccharomyces* yeast where there is some outbreeding, advantageous combinations will be broken up by recombination unless they are linked. This will lead to fixed differences between populations of linked combinations of variants (see Figure 3). Over time several linked fixed variations can be generated between populations, and if any individual variants are initially deleterious, the result will be a linked set of QTLs of mixed effect (positive and negative on the phenotype in question). Different populations adapting to similar niches would likely evolve linked sets in different genomic locations, resulting in the finding of large QTL regions in crosses between these populations which upon further dissection reveal several linked QTLs within each large “super”-QTL. One caveat to this interpretation is that the trait measured may not be the trait acted upon by selection, however the finding of linked QTLs in many examples indicates that the QTLs underlying the traits measured must be somehow interconnected in adaptation. Pleiotropy is likely to be an important aspect of the complex architecture of quantitative traits. As quantitative trait analysis advances in terms of resolution and sensitivity, we predict that this architecture will be found in many situations and is already becoming evident in other systems [29]. The generation and evolution of “supergenes” as seen in complex color patterns in butterflies where recombination is reduced/eliminated between linked genes affecting the phenotype [30] may be analogous to the linked QTLs we see in yeast.

## Next Generation QTL Mapping: From X-QTLs to iQTLs

Multiple aspects and directions are evolving from the conventional QTL mapping approach. The genetic variation so far described in *S. cerevisiae* is potentially only the tip of the iceberg. New natural variants from a wide range of sources and geographic areas have been isolated and genome sequences will soon be available. This reservoir of diversity can aid the bioinformatic analysis for predicting functional variants [31]. An increasing number of sequenced individuals could also enable the use of GWAS. However, the well-defined population structure is likely to pose a problem. Associations within lineages do not suffer from this problem but cannot be applied to many traits that appear characteristic of but do not vary within a population [32]. Choice of mapping approaches depends on the population structure and the nature of the trait in question. Alternative strategies have been worked out in plant systems for different situations [33] varying sampling and breeding strategies for these different situations. QTL mapping in yeast has evolved along these lines outlined for plants with surveys of large numbers of isolates from various sources and locations [13], [34] and individual crosses for specific traits [4], [5], [8], [15], [16], [19], [25]–[27], [35]–[41] to pair-wise crosses between different populations [14], [15], [42]. Sensitivity in detection of QTLs, even those of modest effect, has been enhanced by bulk segregant analysis, either in large numbers of F1 progeny, the X-QTL approach [43], or through large numbers of segregants from advanced intercross lines, the iQTL approach [44]. The advantage of iQTLs (intercross-selection QTLs) over X-QTLs is in resolution as the multiple generations break up linkage groups allowing determination of individual QTLs down to single genes and in some instances the causal QTN ([44] and Figure 2).

GWAS in yeast is likely to be hampered by the population structure, and it is unlikely that enough different mosaic genomes can be sampled from the environment to map more than a few specific QTLs by association. In plants there are already multi-parental, multi-generational strategies [33], which allow for the incorporation of most of the genetic variation of a species into one large panmictic pool, which can then be used for GWAS analysis. An attractive prospect for yeast is to create artificially outbred populations by mixing multiple variants that can then be exploited by new GWAS approaches [44]. This can complement advanced intercross lines created from pairwise crosses [44]. Next generation sequencing allows complete genome-wide genotype data for large segregant populations, particularly important in multigeneration crosses, where the small haplotype block size requires high marker density [44]. Large populations have been used for bulk segregant analysis and successfully mapped variants with minor effect [43]–[46]. The increased ability to synthesize specific DNA sequences is also a promising approach to experimentally measure the effect of specific variants in a high-throughput fashion (Figure 1D, [47]). This should facilitate the assessment of complex interactions, as well as generate synthetic variants that expand the gene pool.

Exploiting natural variation by reverse genetics is also valuable. Deletion collections from highly diverged strains or sister species, such as *S. paradoxus*, allow the testing of whether the observed phenotypes are general or strain background dependent. Initial attempts at predicting phenotypes by combining population genomics with systems biology datasets gave promising results [48]. Characterizing the effects of mutations/deletions in many backgrounds should improve the predictive power.

A further step forward in yeast linkage analysis will be the shift from using haploid (or homozygous diploids) to diploids. This will strengthen the *S. cerevisiae* position as a model for human complex traits and also will provide more realistic insights for yeast in natural settings. Few studies have looked at important aspects such as heterosis and overdominace [49], [50] and heterozygosity [51]. Technological challenges posed by working with diploids appear to have been overcome by new QTL mapping approaches [44]. Going back to Winge's initial experiments, forward genetics in yeast appears to have a bright future ahead and will likely play a key role in elucidating the rules that govern complex traits. In turn, these novel mapping and breeding strategies hold the potential to create attractive *S. cerevisiae* variants for the budding yeast biotechnology industry [28], [35], [38].

These approaches will be useful in other systems. The plant community already has the breeding mapping strategies and they have been very successful [33]. The Drosophila community has successfully used population-based resequencing [52] to map QTLs even with modest effect, and it has a deletion-based testing of candidates analogous to the reciprocal heterozygosity test with a large resource of deletions available [53]. It remains to be seen how complex the quantitative genetic architecture is in other systems compared to yeast, but given the missing heritability in many systems [33], [54], it is likely that the situation in yeast is not unusual.
